# Complete Genome Sequence of Zika Virus from the First Imported Case in Mainland China

**DOI:** 10.1128/genomeA.00291-16

**Published:** 2016-04-21

**Authors:** Lin Liu, Wei Wu, Xiang Zhao, Ying Xiong, Shuo Zhang, Xiaoqing Liu, Jing Qu, Jiandong Li, Kai Nei, Mifang Liang, Yuelong Shu, Guoliang Hu, Xuejun Ma, Dexin Li

**Affiliations:** a NHFPC Key Laboratory for Medical Virology, National Institute for Viral Disease Control and Prevention, China CDC, Beijing, China; b Jiangxi Province CDC, Nanchang, China

## Abstract

The first case of Zika virus infection was identified in a Chinese traveler returning from Venezuela in February 2016. This report describes the complete genome sequence of Zika virus from the first imported case in China. The genome sequence analysis showed that the Zika virus isolated in this case belongs to the Asian lineage.

## GENOME ANNOUNCEMENT

Zika virus (ZIKV) is an emerging mosquito-borne, positive single-stranded RNA virus (1). Only sporadic human infections had been caused by ZIKV in some African and Asian countries until an outbreak occurred in Yap Island in 2007 (2) and later in French Polynesia in 2013 (3). Since May 2015, the disease has rapidly spread in Brazil and in 48 other countries and has caused extensive concern worldwide (4).

The first imported ZIKV case was diagnosed in China. The patient is a 34-year-old Chinese male traveler returning from Caracas, Venezuela, to Ganxian, Jiangxi Province, China, who was admitted to the local hospital on 6 February 2016. He developed fever (38.0°C), headache, and dizziness on 28 January, and rashes, retro-orbital pain, and mild diarrhea later appeared. A quantitative real-time PCR targeting the NS1 gene of ZIKV was developed in-house by the CDC of China. Zika viral RNA was detected in the serum and urine collected from the patient on day 9 post-onset. Viral RNA was extracted from the serum of the patient using the QIAamp viral RNA minikit. PCR products for PGM sequencing were prepared using a one-step RT-PCR system with designed specific primers. A next-generation sequencing library was constructed and analyzed using an Ion Plus fragment library kit (Life Technologies) according to the manufacturer’s instructions and was subsequently sequenced using an Ion PGM Hi-Q OT2 kit on the Ion Torrent PGM platform. The next-generation sequencing data were analyzed with CLC Genomics Workbench version 8.5.1. Phylogenic analysis was performed using MEGA6 program.

For the first imported ZIKV strain, VE_Ganxian, a 10,676-nucleotide (nt)-long consensus contig was produced by 302,215 reads, including 5′-untranslated-region (UTR) (1 to 105 nt), 3′-UTR (10,378 to 10,676 nt), and an open reading frame (ORF) (10,272 nt). The ORF encodes a polyprotein, including three structural proteins, capsid (125 amino acids [aa]), premembrane/membrane (165 aa), and envelope (505 aa); and seven nonstructural proteins: NS1 (362 aa), NS2A (218 aa), NS2B (144 aa), NS3 (603 aa), NS4A (147 aa), NS4B (502 aa), and NS5 (652 aa). Phylogenetic analysis of strain VE_Ganxian and other available genome sequences of ZIKV showed that this strain belongs to the Asian lineage, with highest genetic similarity to strain Haiti/1225/2014 (GenBank no. KU509998) and strain BeH819966 (GenBank no. KU365779), which are dominant sequences from those viruses prevalent and circulating in French Polynesia and Brazil during large outbreaks in 2013 and 2015, respectively. However, VE_Ganxian was originally from Venezuela, and the genome sequence showed 11 aa differences in the E gene and 32 aa differences in the whole genome from strain BeH819966, a Brazilian isolate from 2015.

This is the first time that the complete ZIKV genome sequence from the first imported case of ZIKV infection in China has been reported. It will contribute to a better understanding of the evolution and genetic diversity of ZIKV and can also benefit vaccine development and disease control of Zika fever in China.

### Nucleotide sequence accession number.

The complete genome sequence of strain VE_Ganxian has been deposited in GenBank under the accession number KU744693.
